# Supplementary material for: Prevalence of asymptomatic non-falciparum and falciparum malaria in the 2014-15 Rwanda Demographic Health Survey
Source: PLoS One. 2025 Sep 11;20(9):e0330480. doi: 10.1371/journal.pone.0330480 (PMC12425214; doi:10.1371/journal.pone.0330480)
Supplement: S1 Table — Malaria speciation qPCR assay details. (PDF) [file pone.0330480.s003.pdf]

S1 Table. PCR Primers, Probes and Reaction Conditions. Malaria speciation qPCR assay details.

Plasmodium falciparum (varATS)

Adapted from: Hoffman, N, et. al. PLOS Medicine. 2015.

Forward Primer 5'- CCCATACACAACCAAYTGGA - 3'
Reverse Primer 5' - TTCGCACATATCTCTATGTCTATCT - 3'
Probe 5' - 6-FAM-TRTTCCATAAATGGT-NFQ-MGB - 3'

Cycling conditions			*45 cycles*	
temp (degrees Celsius)	50	95	95	55
time	2 min	10 min	15 s	1 min

Roche FastStart Universal Probe Master	
Fwd primer	800 nM
Rev primer	800 nM
Probe	400 nM
Template DNA	2.5 µl
Total volume	12.5 µl

Plasmodium malariae (18s)

Rougemont M, et. al. Journal of Clinical Microbiology. 2004.

5' - AGTTAAGGGAGTGAAGACGATCAGA - 3'
5' - CAACCCAAAGACTTTGATTTCTCATAA - 3'
5' - 6-FAM-ATGAGTGTTTCTTTTAGATAGC-NFQ-MGB - 3'

			45 cycles	
	50	95	95	60
	2 min	10 min	15 s	1 min

Roche FastStart Universal Probe Master	
Fwd primer	300 nM
Rev primer	300 nM
Probe	400 nM
Template DNA	2 µl
Total volume	12.5 µl

Plasmodium ovale (18s)

Adapted from: Mitchell C, et. al. Journal of Infectious Diseases. 2021

Forward Primer 5' - CCRACTAGGTTTTGGATGAAAVRTTTTT- 3'
Reverse Primer 5' - AACCCAAAGACTTTGATTTCTCATAA - 3'
Probe 5' - VIC/CRAAAGGAATTYTCTTATT - 3'

Cycling conditions			*45 cycles*	
temp (degrees Celsius)	50	95	95	52
time	2 min	10 min	15 s	1 min

Roche FastStart Universal Probe Master (Rox)	
Fwd primer	400 nM
Rev primer	400 nM
Probe	200 nM
Template DNA	2 µl
Total volume	12 µl

Plasmodium vivax (18s)

Brazeau N, et. al. Nature Communications. 2021

5' - ACGCTTCTAGCTTAATCCACATAACT - 3'
5' - ATTTACTCAAAGTAACAAGGACTTCCAAGC - 3'
5' - /56-FAM/TTCGTATCG/ZEN/ACTTTGTGCGCATTTTGC/3IABkFQ/ - 3'

			45 cycles	
	50	95	95	60
	2 min	10 min	15 s	1 min

Roche FastStart Universal Probe Master (Rox)	
Fwd primer	400 nM
Rev primer	400 nM
Probe	200 nM
Template DNA	5 µl
Total volume	18 µl
