# Supplementary material for: Prevalence of asymptomatic non-falciparum and falciparum malaria in the 2014-15 Rwanda Demographic Health Survey
Source: PLoS One. 2025 Sep 11;20(9):e0330480. doi: 10.1371/journal.pone.0330480 (PMC12425214; doi:10.1371/journal.pone.0330480)
Supplement: S2 Table — Each assay was run to 45 cycles against plasmids diluted to representative parasitemias (based on 6 copies per genomic equivalent). (PDF) [file pone.0330480.s004.pdf]

S2 Table. Cross reactivity of non-falciparum real time PCRs at 45 cycles. Each assay was run to 45 cycles against plasmids diluted to representative parasitemias (based on 6 copies per genomic equivalent).

Assay	***Template***	***Genomic equivalent (GE)***	***# positive***	***CT of positive***
P. vivax	*P. falciparum*	212 GE/µL	0/20	NA
	P. ovale	110 GE/µL	0/20	NA
	P. malariae	100 GE/µL	1/20	42.1
P. ovale	*P. falciparum*	212 GE/µL	1/20	43.7
	P. vivax	76.5 GE/µL	0/20	NA
	P. malariae	100 GE/µL	1/20	42.5
P. malariae	*P. falciparum*	212 GE/µL	0/20	NA
	P. vivax	76.5 GE/µL	0/20	NA
	P. ovale	110 GE/µL	0/20	NA
