# Supplementary material for: Prevalence of asymptomatic non-falciparum and falciparum malaria in the 2014-15 Rwanda Demographic Health Survey
Source: PLoS One. 2025 Sep 11;20(9):e0330480. doi: 10.1371/journal.pone.0330480 (PMC12425214; doi:10.1371/journal.pone.0330480)
Supplement: S3 Table — Weighted counts in each category of analysis variables, for both the study population and all participants with DBS collected for HIV testing in the 2014−15 DHS. (PDF) [file pone.0330480.s005.pdf]

S3 Table. Comparison of Population Used for Molecular Screening to Overall DHS Population. Weighted counts in each category of analysis variables, for both the study population and all participants with DBS collected for HIV testing in the 2014-15 DHS.

Variable		*This Study*	*%*	*Total DHS HIV DBS*	*%*
Sex of respondent	Male	2490	48.84%	8314	49.02%
	Female	2609	51.16%	8645	50.98%
Age group (years)	0-14	77	1.52%	230	1.36%
	15-24	724	14.20%	4363	25.73%
	25-34	1651	32.37%	4758	28.05%
	35-44	1449	28.42%	4138	24.40%
	45-54	804	15.77%	2340	13.80%
	55+	394	7.73%	1129	6.66%
Wealth Quintile	Poorest	744	14.59%	2891	17.05%
	Poorer	897	17.59%	3245	19.14%
	Middle	917	17.98%	3418	20.16%
	Richer	1103	21.63%	3562	21.01%
	Richest	1439	28.21%	3841	22.65%
Education	None/preschool	640	12.55%	3262	19.23%
	Primary	3191	62.58%	10420	61.45%
	Secondary	1073	21.05%	2803	16.53%
	Higher	192	3.76%	456	2.69%
	Missing data	3	0.06%	17	0.10%
Owns livestock, herds, or farm animals	No	2199	43.13%	7279	42.92%
	Yes	2900	56.87%	9679	57.08%
Source of drinking water	Unpiped	4321	84.73%	15080	88.92%
	Piped	779	15.27%	1879	11.08%
Household bed net	No	904	17.72%	2802	16.52%
	Yes	4196	82.28%	14157	83.48%
Slept under LLIN last night	No	1926	37.78%	6367	37.54%
	Yes	3173	62.22%	10592	62.46%
Insecticide-treated household net	No	3	0.06%	20	0.11%
	Yes	3175	62.26%	10603	62.52%
	Missing data	1921	37.68%	6336	37.36%
1 bed net per 1.8 household members	No	3870	76.04%	13426	79.34%
	Yes	1219	23.96%	3497	20.66%
Cluster level covariates					
Region	Kigali City	834	16.36%	2156	12.71%

	South	1117	21.90%	4075	24.03%
	West	1300	25.49%	3768	22.22%
	North	1066	20.91%	2723	16.06%
	East	782	15.34%	4237	24.98%
Place of residence	Urban	1239	24.30%	3163	18.65%
	Rural	3860	75.70%	13796	81.35%
Elevation (m)	500-1000	17	0.34%	62	0.36%
	1001-1500	1234	24.21%	5036	29.70%
	1501-2000	2631	51.60%	8914	52.56%
	2001-2500	1108	21.73%	2798	16.50%
	2500 <	108	2.11%	149	0.88%
Month of data collection	15-Jan	1377	27.01%	4004	23.61%
	15-Feb	890	17.45%	3019	17.80%
	15-Mar	1031	20.22%	3628	21.40%
	15-Apr	43	0.85%	107	0.63%
	14-Nov	595	11.67%	2426	14.30%
	14-Dec	1163	22.80%	3775	22.26%
Land cover	Moderate forest	316	6.19%	960	5.66%
	Sparse forest	44	0.87%	263	1.55%
	Woodland	821	16.09%	3260	19.23%
	Closed grassland	3434	67.34%	11253	66.36%
	Perennial cropland	485	9.51%	1222	7.21%
