# Supplementary material for: Prevalence of asymptomatic non-falciparum and falciparum malaria in the 2014-15 Rwanda Demographic Health Survey
Source: PLoS One. 2025 Sep 11;20(9):e0330480. doi: 10.1371/journal.pone.0330480 (PMC12425214; doi:10.1371/journal.pone.0330480)
Supplement: S4 Table — 3/160 replicates were false positive across assays: 1.9% [95% CI: 0.3–5.4] Binomial exact method. All adjustments rounded up to the higher integer. (PDF) [file pone.0330480.s006.pdf]

S4 Table. Impact of potential false positivity on unadjusted prevalence. 3/160 replicates were false positive across assays: 1.9% [95% CI: 0.3-5.4] Binomial exact method. All adjustments rounded up to the higher integer.

Number tested		*# CT ≤ 40*	*# CT >40*	*Unweighted prevalence*	*0.3% false positivity among CT >40*	*Adjusted unweighted prevalence*	*5.4% false positivity among CT>40*	*Adjusted unweighted prevalence*
5,050	*P. falciparum*	1297	181	29.3%	180	29.3%	171	29.1%
5,050	*P. malariae*	159	27	3.7%	27	3.7%	26	3.7%
5,050	*P. ovale*	124	159	5.6%	159	5.6%	150	5.4%
