# Supplementary material for: Prevalence of asymptomatic non-falciparum and falciparum malaria in the 2014-15 Rwanda Demographic Health Survey
Source: PLoS One. 2025 Sep 11;20(9):e0330480. doi: 10.1371/journal.pone.0330480 (PMC12425214; doi:10.1371/journal.pone.0330480)
Supplement: S5 Table — Change in district level prevalence using only PCR positive infections with CT values 40 or less, compared to 45 cycles. (PDF) [file pone.0330480.s007.pdf]

S5 Table. Differences in District Level Prevalence by PCR Cutoff. Change in district level prevalence using only PCR positive infections with CT values 40 or less, compared to 45 cycles.

District	*malaria_prevΔ*	*pf_prevΔ*	*pm_prevΔ*	*po_prevΔ*	*pv_prevΔ*
Nyarugenge	0.114	0.037	0.025	0.052	0.000
Gatsibo	0.105	0.040	0.000	0.104	0.000
Ngoma	0.099	0.058	0.002	0.087	0.001
Burera	0.091	0.043	0.020	0.028	0.000
Huye	0.090	0.073	0.002	0.037	0.000
Gasabo	0.084	0.032	0.000	0.052	0.000
Rwamagana	0.084	0.052	0.014	0.063	0.000
Kirehe	0.082	0.052	0.002	0.043	0.000
Kayonza	0.074	0.060	0.000	0.021	0.000
Ruhango	0.072	0.026	0.030	0.046	0.000
Kamonyi	0.065	0.026	0.007	0.047	0.000
Karongi	0.063	0.001	0.000	0.063	0.000
Nyamagabe	0.060	0.043	0.000	0.026	0.000
Rubavu	0.059	0.028	0.000	0.031	0.000
Gakenke	0.058	0.023	0.036	0.000	0.000
Nyaruguru	0.055	0.043	0.000	0.033	0.000
Muhanga	0.055	0.019	0.000	0.036	0.000
Nyabihu	0.055	0.021	0.009	0.017	0.008
Gisagara	0.054	0.050	0.002	0.072	0.000
Gicumbi	0.050	0.019	0.007	0.024	0.000
Kicukiro	0.044	0.033	0.000	0.018	0.000
Nyagatare	0.042	0.055	0.000	0.023	0.000
Nyanza	0.037	0.036	0.005	0.016	0.000
Nyamasheke	0.036	0.036	0.000	0.000	0.000
Rutsiro	0.034	0.042	0.000	0.000	0.012
Musanze	0.033	0.017	0.007	0.009	0.000
Bugesera	0.032	0.015	0.001	0.021	0.006
Rusizi	0.017	0.008	0.009	0.000	0.000
Rulindo	0.015	0.008	0.000	0.007	0.000
Ngororero	0.015	0.007	0.008	0.008	0.000
