# Supplementary material for: Prevalence of asymptomatic non-falciparum and falciparum malaria in the 2014-15 Rwanda Demographic Health Survey
Source: PLoS One. 2025 Sep 11;20(9):e0330480. doi: 10.1371/journal.pone.0330480 (PMC12425214; doi:10.1371/journal.pone.0330480)
Supplement: S6 Table — Weighted n denotes the total weighted number for participants in each cluster, while all other values are district level prevalences. (PDF) [file pone.0330480.s008.pdf]

S6 Table. District Level Malaria Prevalence at Different Cycle Cutoffs. Weighted n denotes the total weighted number for participants in each cluster, while all other values are district level prevalences.

		45 cycles						***40 cycles***					
District	*weighted n*	*all malaria*	*non-Pf*	*Pf*	*Pm*	*Po*	*Pv*	*all malaria*	*non-Pf*	*Pf*	*Pm*	*Po*	*Pv*
Bugesera	90.1	32.6	5.2	29.9	1.5	3.4	0.6	29.7	2.6	28.5	1.3	1.5	0.0
Burera	219.5	35.2	21.0	16.3	9.2	11.8	0.0	15.2	10.5	6.8	4.9	5.7	0.0
Gakenke	189.2	14.2	8.7	5.4	8.7	0.0	0.0	3.2	2.0	1.1	2.0	0.0	0.0
Gasabo	391.1	76.2	20.5	55.7	0.0	20.5	0.0	43.3	0.0	43.3	0.0	0.0	0.0
Gatsibo	110.2	40.0	15.5	30.4	0.6	14.9	0.0	28.4	4.1	26.0	0.6	3.5	0.0
Gicumbi	228.0	30.7	18.5	12.2	13.0	5.5	0.0	19.3	11.5	7.9	11.5	0.0	0.0
Gisagara	129.9	64.5	18.4	59.2	3.9	14.7	1.1	57.5	9.0	52.7	3.6	5.4	1.1
Huye	117.5	56.8	15.2	51.4	0.2	15.0	0.0	46.2	10.6	42.9	0.0	10.6	0.0
Kamonyi	137.3	35.2	10.5	27.9	1.5	9.0	0.0	26.2	3.1	24.3	0.6	2.6	0.0
Karongi	228.8	36.3	22.9	15.7	3.6	19.3	0.0	21.8	8.4	15.4	3.6	4.8	0.0
Kayonza	108.2	39.0	7.2	33.2	2.1	5.1	0.0	30.9	4.9	26.6	2.1	2.8	0.0
Kicukiro	213.9	29.9	13.3	17.8	8.9	5.7	0.0	20.5	10.8	10.8	8.9	1.9	0.0
Kirehe	102.8	60.6	13.8	53.7	5.9	8.4	0.0	52.1	9.5	48.4	5.7	4.0	0.0
Muhanga	158.0	26.1	7.3	20.5	0.9	6.4	0.0	17.4	1.7	17.4	0.9	0.8	0.0
Musanze	242.4	22.8	12.2	10.6	9.9	2.3	0.0	14.8	8.4	6.4	8.4	0.0	0.0
Ngoma	119.8	68.5	24.6	57.6	8.3	17.6	0.2	56.6	14.7	50.7	8.0	7.1	0.0
Ngororero	210.0	19.3	13.1	7.6	10.3	4.5	0.0	16.2	11.4	6.1	8.6	2.9	0.0
Nyabihu	200.1	21.3	15.4	6.8	9.4	4.4	1.6	10.3	8.6	2.6	7.6	1.0	0.0
Nyagatare	144.1	35.3	14.6	26.9	10.3	4.4	0.0	29.2	11.4	19.0	10.3	1.1	0.0
Nyamagabe	203.5	53.8	19.4	37.2	10.0	9.5	0.0	41.7	14.1	28.5	10.0	4.1	0.0
Nyamasheke	144.9	26.7	4.8	21.9	4.8	0.0	0.0	21.5	4.8	16.7	4.8	0.0	0.0
Nyanza	126.3	69.2	12.6	65.8	6.2	6.8	0.0	64.5	10.3	61.3	5.7	4.8	0.0
Nyarugenge	229.5	41.7	21.2	20.6	8.1	13.9	1.6	15.6	3.5	12.0	2.4	1.9	1.6

Nyaruguru	108.1	27.0	9.5	23.3	4.0	5.5	0.0	21.0	5.9	18.6	4.0	1.9	0.0
Rubavu	202.9	32.8	13.7	19.1	3.9	9.9	0.0	20.8	7.4	13.3	3.9	3.6	0.0
Ruhango	136.0	48.6	14.7	39.1	6.6	9.1	0.0	38.7	5.4	35.5	2.5	2.9	0.0
Rulindo	187.0	19.0	4.8	14.2	1.4	3.3	0.0	16.1	3.4	12.8	1.4	2.0	0.0
Rusizi	180.1	24.0	9.5	15.1	9.5	0.0	0.0	21.0	7.9	13.7	7.9	0.0	0.0
Rutsiro	132.9	13.8	5.8	9.1	4.7	1.1	1.6	9.2	5.8	3.4	4.7	1.1	0.0
Rwamagana	107.1	35.6	12.6	27.2	5.0	8.5	0.7	26.6	5.9	21.6	3.4	1.7	0.7
